# Ileorectal intussusception in an adult: a case report of an anal mass

**DOI:** 10.1093/gastro/goad067

**Published:** 2023-10-31

**Authors:** Azin Kalani, Mina Alvandipour

**Affiliations:** Department of Surgery, Faculty of Medicine, Mazandaran University of Medical Sciences, Sari, Mazandaran, Iran; Department of Surgery, Faculty of Medicine, Mazandaran University of Medical Sciences, Sari, Mazandaran, Iran

## Introduction

Intussusception happens when one part of the intestine invaginates into another adjacent part [1]. Transanal protrusion of intussusception, which is also known as intussusception prolapse, is described as the protrusion of the top of the intussusception from the anus. This presentation accounts for 8%–29% of intussusceptions [2–4]. Intussusception is rare in adults and commonly occurs as a complication for patients with organic diseases. Our case is a transanal protrusion of intussusception, which has occurred in the ileorectal anastomosis site. This makes the case unique.

## Case report

**Figure 1.**
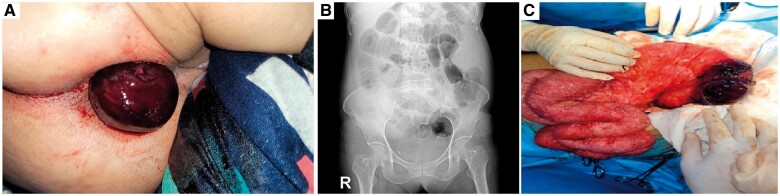
The clinical data of the case with ileorectal intussusception. (A) The anal mass. (B) Abdominal X-ray imaging showing the obstruction. (C) Reduction of the protruding anal mass. Necrosis of the protruding mass was seen.

The patient was a 62-year-old female who presented with the main complaint of an anal mass protruding after defecation and ∼6 hours before hospitalization. She did not have any nausea or vomiting. The patient mentioned a history of occasional abdominal pain, which did not lead to hospitalization. Physical examination revealed mild tenderness in the low abdomen. A bulky mass protruded through the rectum mimicking prolapse on rectal examination. The anal mass was engorged and painful (Figure 1A). Due to a neoplasm, she had a total colectomy and ileorectal anastomosis 10 years ago. Left mastectomy was performed 5 years ago. The patient also gave a suspicious history of pelvic radiotherapy due to bone metastasis 5 years ago but unfortunately she did not bring any documentary evidence. Vital signs were abnormal: blood pressure 85/65 mmHg, pulse rate 110/min, temperature 38°C, respiration 21/min. Laboratory tests determined the leukocytosis (white blood cell count 21.1 × 10^9^/L) and metabolic acidosis combined with respiratory acidosis (arterial blood gas: pH 7.22, PCO_2_ 46.3 mmHg, HCO_3_^−^ 16.2 mmol/L). The patient's liver and kidney function were normal. In addition, radiological imaging showed an obstruction (Figure 1B).

The patient was initially treated with antibiotics and intravenous fluids, and then she was scheduled for laparotomy. The mass was checked again in the operating room and the protruding mass seemed to be a small intestine. The laparotomy was performed with a low midline incision. After adhesiolysis and evaluation, an intussusception was found at the previous anastomosis site. Reduction of the protruding anal mass was then performed and 15 cm of the terminal ileum was found protruding through the ileorectal anastomosis into the rectal stump (Figure 1C). Due to the severe inflammation, the gangrenous intestine was resected and an end ileostomy was performed.

## Discussion

Intussusception is an uncommon condition in adults. The accurate mechanism for intestine intussusception has not yet been realized appropriately. In most previous reports in adults, any intraluminal irritation or lesion in the intestinal wall may alter intestinal peristalsis, thereby causing intestinal invagination [5]; the main cause is usually a pathological leading point such as a polyp or malignancy in the gastrointestinal system, accounting for 66% of colonic intussusceptions and 30% of small bowel intussusceptions [6, 7]. Of these, only 1%–4% occur in the anastomotic site [8, 9]. Hayama *et al.* [10] reported a case in which intussusception developed after laparoscopic right hemicolectomy at the ileocolic anastomosis site.

Several hypotheses, including extensive non-gentle handling and intestinal drying during primary surgery, prolonged post-operative ileus, and post-operative chemotherapy or radiation, are suggested for the pathogenetic mechanisms of post-operative intussusception. In the present case, the patient’s intestines had severe adhesions, which may have been caused by the history of total colectomy surgery and radiotherapy. Moreover, widening the ileorectal anastomosis site may be a key factor in intussusception. Anastomosis is a functional end-to-end process that theoretically might have a higher chance of this complication where one end is closed off around an area of sharp angulation; angulation with excessive asymmetric adhesions may potentially lead to an angulated peristalsis of the bowel, which could encourage one side to become the leading point of the intussusceptum. Furthermore, no polyp or true mass was detected in our case.

A protruded mass occurs in transanal intussusception and rectal prolapse. Nonetheless, it is possible to differentiate between intussusception and prolapse by patient history, physical examination, and further investigations. One distinction is the presence of a leading point in intussusception. Another way to differentiate is by using digital examination. The anal crypts are everted in rectal prolapse but not in intussusception; in patients with intussusception, the examination finger can be passed between the anus and the protruding mass, whereas this is impossible in patients with rectal prolapse. In addition, full-thickness rectal prolapse occurs when part of the rectal wall protrudes from the anus; the protrusion of the rectal lining from the anus leads to mucosa prolapse. Rectal internal intussusception occurs when the rectum is pulled inward without protruding from the anus.

In conclusion, intussusception in adults is a rare pathology with a challenging diagnosis that requires a high degree of skepticism. In addition to abnormalities of the intestinal wall, including polyps or tumors, as the main causes of intussusception in adults, we should also consider anastomotic intussusception in patients with previous anastomosis in the gastrointestinal system, similarly to our patient.

## Authors’ Contributions

A.K. and M.A. designed this study. A.K. collected the information and images, and wrote the manuscript. M.A. reviewed the manuscript. Both authors read and approved the final version of the manuscript.
